# Dose-response relationships of sand training compared to other surface training in improving change of direction and jump performance: a systematic review and meta-analysis

**DOI:** 10.3389/fphys.2025.1737074

**Published:** 2026-02-09

**Authors:** Tingting Wang, Haiting Zhai, Hao Yan, Yuping Zhou, Zhi Li, Hongwen Wei, Qian Geng

**Affiliations:** 1 Beijing Sport Science Institute, Beijing, China; 2 Key Laboratory for Performance Training and Recovery of General Administration of Sport, Beijing Sport University, Beijing, China; 3 College of Strength and Conditioning Training, Beijing Sport University, Beijing, China; 4 Sports Coaching College, Beijing Sports University, Beijing, China; 5 Naval Aviation University, Yantai, China; 6 Zhejiang College of Construction, Hangzhou, China; 7 University of Macau, Macao, China; 8 School of Continuous Education, Beijing Sport University, Beijing, China

**Keywords:** change of direction, firm, sand, standing long jump, surface training, systematic review

## Abstract

**Objective:**

Change of direction (COD) and jump performance are critical for success in many sports. Sand training, utilizing an unstable surface, is believed to improve these abilities, but its effectiveness compared to hard-surface training remains unclear. This study aims to compare the effects of sand training versus hard-surface training on COD and jump performance.

**Methods:**

A systematic search was conducted across PubMed, MEDLINE, CENTRAL, Scopus, and Web of Science databases. Data were analyzed using Stata 15 and RevMan. The quality of the included studies was assessed using the PEDro scale.

**Results:**

Fourteen studies met the inclusion criteria. No publication bias was detected (p > 0.05). Sand training showed greater effectiveness than hard-surface training in the T-test (SMD = −0.80, p = 0.04) and standing long jump (SLJ) (SMD = 0.85, p = 0.004). For the T-test, training programs longer than 6 weeks (SMD = −1.19, p = 0.007), with 3 sessions per week (SMD = −1.15, p = 0.01), and sessions lasting less than 40 min (SMD = −1.10, p = 0.01) yielded better results. For SLJ, programs lasting over 6 weeks (SMD = 1.42, p = 0.05) with more than 3 sessions per week (SMD = 1.04, p = 0.003) were more effective. Trained individuals showed greater improvements in the T-test (SMD = −1.44, p = 0.002), while untrained individuals demonstrated more significant improvements in the SLJ (SMD = 0.68, p = 0.005).

**Conclusion:**

Sand training enhances COD ability and horizontal jump performance more effectively than training on hard surfaces. No significant differences were found between surfaces for countermovement jump or squat jump. For COD, training programs exceeding 6 weeks, with 3 weekly sessions lasting under 40 min, yielded better outcomes. For horizontal jump performance, training with at least 3 sessions per week for over 6 weeks proved most effective. Trained individuals benefitted more in COD ability, whereas untrained individuals saw greater improvement in horizontal jump performance.

**Systematic Review Registration:**

Identifier CRD420251160919.

## Introduction

Change of direction (COD) and jumping ability are fundamental for performance in high-intensity team sports like soccer, basketball, and rugby (Sheppard and Young, 2006). These sports require the repeated execution of rapid movements, such as abrupt stops, explosive accelerations, turns, and jumps (Makaruk et al., 2020).

COD ability is defined as a change in movement direction performed in the absence of a reaction to an external stimulus, representing a predetermined movement pattern (Sheppard and Young, 2006). According to the universal agility model, this ability is collectively influenced by leg muscle qualities, straight sprinting speed and movement technique (Young et al., 2002). Specifically, superior concentric strength and power are required to facilitate explosive reacceleration (Young et al., 2002). Simultaneously, reactive strength is heavily relied upon to optimize the efficiency of the stretch-shortening cycle (SSC) during the braking phase (Young et al., 2002). Furthermore, from a technical perspective, eccentric braking is utilized to actively lower the center of gravity and adjust stride patterns, thereby effectively controlling body inertia to execute the directional change (Sayers, 2000).

Jump performance serve as a key indicator of SSC function. Based on ground contact time, SSC is classified into slow (>250 mm) and fast (<250 mm) types (Schmidtbleicher, 1992). Typically, fast SSC is assessed using the drop jump, whereas typical assessments for slow SSC include the countermovement jump (CMJ), squat jump (SJ) and standing long jump (SLJ) (Sc and hmidtbleicher, 1987; Schmidtbleicher, 1992). Characterized by long ground contact time and large joint angular displacements, slow SSC relies primarily on active muscle contraction and adequate cross bridge formation to maximize force (Bobbert et al., 1996; Turner and Jeffreys, 2010). In contrast, fast SSC performance is predominantly determined by tendon stiffness and the recoil of elastic potential energy (Aeles and Vanwanseele, 2019; Walshe et al., 1998). Furthermore, this mechanism relies heavily on neuromuscular pre-activation and co-contraction strategies to optimize movement efficiency (Aeles and Vanwanseele, 2019).

Plyometric, strength, sprint, and sport-specific agility training are established methods for improving COD ability and jump performance (Asadi et al., 2016; Nygaard Falch et al., 2019; Sun et al., 2025). Nygaard Falch et al. demonstrated correlations ranging from moderate to large (r = 0.3–0.9) between these training modalities and COD outcomes, confirming their effectiveness (Nygaard Falch et al., 2019). In particular, plyometric and strength training yielded the most pronounced improvements in drop jump and CMJ (Nygaard Falch et al., 2019; Peterson et al., 2006). Specifically, plyometric training has been shown to enhance COD capabilities dominated by both force and velocity (Nygaard Falch et al., 2019). Moreover, these interventions optimize performance by inducing increased muscle strength, enhanced motor unit recruitment, improved intermuscular coordination, and augmented efficiency of the SSC (Nim et al., 2010; Young and Farrow, 2006). However, despite being a critical factor, the potential moderating role of the training surface on training efficacy has been largely overlooked in current literature.

Then distinct physiological and biomechanical differences exit during exercise on different training surfaces (Lejeune et al., 1998; Pinnington and Dawson, 2001; Strydom et al., 1966; Zamparo et al., 1992). These differences alter energy expenditure and neuromuscular recruitment patterns in athletes (Binnie et al., 2013; Pinnington et al., 2005; Strydom et al., 1966), thereby eliciting unique stimuli and adaptive changes.

For instance, compared to stable ground, the instability and compliance of sand surfaces result in reduced elastic energy utilization, decreased efficiency of the muscle tendon unit, and increased mechanical work (Zamparo et al., 1992). This leads to greater energy expenditure (Pinnington and Dawson, 2001). Pereira et al. proposed that sand training increases the activation magnitude of target muscles during movement, thereby enhancing neuromuscular performance (Pereira et al., 2021). Concurrently, Hammami et al. demonstrated that plyometric training on sand enhances nerve conduction velocity, motor unit recruitment, and Hoffmann reflex excitability (Hammami et al., 2020). These adaptations effectively improve neuromuscular function (Hammami et al., 2020). Furthermore, studies indicate that shock absorptive capacity of sand attenuates impact forces on soft tissues and bones compared to rigid surfaces, such as wooden floors or grass (Mirzaei et al., 2014; Miyama and Nosaka, 2004). This attenuation decreases muscle soreness and injury risk, offering potential value during preseason and rehabilitation periods (Mirzaei et al., 2014; Miyama and Nosaka, 2004).

However, evidence regarding the superiority of sand training over other surfaces remains inconsistent. Impellizzeri et al. observed that plyometric training on both sand and grass improved sprint performance. For jump performance, grass training significantly enhanced CMJ height, whereas sand training elicited greater improvements in SJ (Impellizzeri et al., 2008). Conversely, a meta-analysis by Pereira er al. Reported that while sand training effectively improved jump and sprint abilities, its efficacy was similar to that of rigid surfaces (Pereira et al., 2021). In fact, researches indicate that compliant sand surfaces significantly reduce the efficiency of the SSC, thereby limiting speed and power performance (Binnie et al., 2014; Pereira et al., 2021). This limitation occurs because sand dissipates more energy upon landing compared to rigid surfaces, resulting in reduced stride length and horizontal velocity (Alcaraz et al., 2011). Concurrently, surface instability attenuates the myotatic reflex upon landing, reduces the storage and reuse of elastic energy, and prolongs the amortization phase, diminishing the potentiation effect of the SSC (de Villarreal et al., 2024; Impellizzeri et al., 2008; Singh et al., 2014). Collectively, conclusions regarding the impact of different training surfaces on athletic performance remain inconclusive. These discrepancies likely stem from multifactorial differences in training protocols, including load, frequency and duration. Variables that require systematic quantification and comparison. Furthermore, the inclusion of non-randomized studies in previous reviews may have compromised the reliability of their conclusions.

The dose-response relationship between sand training and athletic performance requires further investigation. Therefore, this study aims to: (a) systematically evaluate the effects of sand versus firm-ground training on COD ability and jump performance via meta-analysis; (b) assess dose-response relationships through subgroup analysis of training volume; and (c) develop evidence-based guidelines for sand training prescription.

## Methods

This systematic review and meta-analysis followed the 2020 PRISMA guidelines (Table 1). The protocol was prospectively registered in the PROSPERO database (ID: CRD420251160919).

**TABLE 1:** PRISMA 2020 checklist.

Section/topic	#	Checklist item	Reported on page #
Title			
Title	1	Identify the report as a systematic review, meta-analysis, or both	Page 1
Abstract			
Structured summary	2	Provide a structured summary including, as applicable: background; objectives; data sources; study eligibility criteria, participants, and interventions; study appraisal and synthesis methods; results; limitations; conclusions and implications of key findings; systematic review registration number	Page 1
Introduction			
Rationale	3	Describe the rationale for the review in the context of what is already known	Page 2
Objectives	4	Provide an explicit statement of questions being addressed with References to participants, interventions, comparisons, outcomes, and study design (PICOS)	Page 2
Methods			
Protocol and registration	5	Indicate if a review protocol exists, if and where it can be accessed (e.g., web address), and, if available, provide registration information including registration number	Page 2
Eligibility criteria	6	Specify study characteristics (e.g., PICOS, length of follow-up) and report characteristics (e.g., years considered, language, publication status) used as criteria for eligibility, giving rationale	Page 2–3
Information sources	7	Describe all information sources (e.g., databases with dates of coverage, contact with study authors to identify additional studies) in the search and date last searched	Page 2–3
Search	8	Present full electronic search strategy for at least one database, including any limits used, such that it could be repeated	Page 2–3
Study selection	9	State the process for selecting studies (i.e., screening, eligibility, included in systematic review, and, if applicable, included in the meta-analysis)	Page 2–3
Data collection process	10	Describe method of data extraction from reports (e.g., piloted forms, independently, in duplicate) and any processes for obtaining and confirming data from investigators	Page 3
Data items	11	List and define all variables for which data were sought (e.g., PICOS, funding sources) and any assumptions and simplifications made	Page 3
Risk of bias in individual studies	12	Describe methods used for assessing risk of bias of individual studies (including specification of whether this was done at the study or outcome level), and how this information is to be used in any data synthesis	Page 3
Summary measures	13	State the principal summary measures (e.g., risk ratio, difference in means)	Page 3
Synthesis of results	14	Describe the methods of handling data and combining results of studies, if done, including measures of consistency (e.g., I^2^) for each meta-analysis	Page 3
Risk of bias across studies	15	Specify any assessment of risk of bias that may affect the cumulative evidence (e.g., publication bias, selective reporting within studies)	Page 3
Additional analyses	16	Describe methods of additional analyses (e.g., sensitivity or subgroup analyses, meta-regression), if done, indicating which were pre-specified	Page 3
Results			
Study selection	17	Give numbers of studies screened, assessed for eligibility, and included in the review, with reasons for exclusions at each stage, ideally with a flow diagram	Page 3
Study characteristics	18	For each study, present characteristics for which data were extracted (e.g., study size, PICOS, follow-up period) and provide the citations	Page 3–4
Risk of bias within studies	19	Present data on risk of bias of each study and, if available, any outcome level assessment (see item 12)	Page 4
Results of individual studies	20	For all outcomes considered (benefits or harms), present, for each study: (a) simple summary data for each intervention group (b) effect estimates and confidence intervals, ideally with a forest plot	Page 4
Synthesis of results	21	Present results of each meta-analysis done, including confidence intervals and measures of consistency	Page 4–5
Risk of bias across studies	22	Present results of any assessment of risk of bias across studies (see item 15)	Page 4–5
Additional analysis	23	Give results of additional analyses, if done (e.g., sensitivity or subgroup analyses, meta-regression [see item 16])	Page 4–5
Discussion			
Summary of evidence	24	Summarize the main findings including the strength of evidence for each main outcome; consider their relevance to key groups (e.g., healthcare providers, users, and policymakers)	Page 5
Limitations	25	Discuss limitations at study and outcome level (e.g., risk of bias), and at review-level (e.g., incomplete retrieval of identified research, reporting bias)	Page 6–7
Conclusions	26	Provide a general interpretation of the results in the context of other evidence, and implications for future research	Page 8
Funding			
Funding	27	Describe sources of funding for the systematic review and other support (e.g., supply of data); role of funders for the systematic review	Page 8

### Search strategy

Literature searches were conducted in five databases—PubMed, MEDLINE, CENTRAL, Scopus, and Web of Science—up to 31 October 2025. Search terms were derived from existing literature and the study’s objectives, combined using Boolean operators (AND/OR) across four conceptual categories: (“Training” OR “Sprint Training” OR “Plyometric Training” OR “Physical Training” OR “Sand Exercise”) AND (“Sand” OR “Beach”) AND (“Jump Height” OR “Jump Distance” OR “Countermovement Jump” OR “CMJ” OR “Squat Jump” OR “SJ” OR “Standing Long Jump” OR “SLJ” OR “Change Of Direction” OR “Agility” OR “T-Test” OR “Illinois Agility Test”) AND (“Healthy People” OR “Adults” OR “Athletes” OR “Adolescents” OR “Children” OR “Young People”).

### Data extraction

Two independent reviewers (T.T.W. and H.T.Z.) extracted data using a standardized form. The extracted data included post-intervention means and standard deviations, as well as study characteristics such as basic information (authors, publication year, sample size), intervention parameters (type, weekly frequency, total duration), participant demographics (sex, age, training background), and outcome measures (jump tests: CMJ, SJ, SLJ; COD: T-Test). Any discrepancies in data extraction were resolved through consultation with a third reviewer (H.Y.).

### Inclusion and exclusion criteria

Study eligibility was determined based on the PICOS framework. The specific inclusion and exclusion criteria are outlined in Table 2.

**TABLE 2:** Study inclusion and exclusion criteria.

Category	Inclusion criteria	Exclusion criteria
Population	Healthy adults, adolescents, children, and athletes	Older adults and unhealthy populations
Intervention	Training interventions (e.g., plyometrics, sprint training) with a minimum duration of 4 weeks	Acute interventions or training programs lasting than 4 weeks
Comparator	The experimental group performs training on a sand surface; the control group performs identical training on a non – sand surface (e.g., grass, court)	Studies without control group or comparable surface – based training
Outcome	Assessments of jump height/distance and change of direction ability	Missing post – intervention data or unavailable outcome measures
Study design	Randomized controlled trials (RCTs)	Case reports, animal studies, reviews and conference abstracts

### Risk of bias assessment

Methodological quality and risk of bias were assessed using the PEDro scale (Nakagawa et al., 2017). Two reviewers independently rated each study, and disagreements were resolved by consensus with a third reviewer. The first item of the PEDro scale was excluded from the total score. A total score of six or higher was considered to indicate high methodological quality.

### Statistical analysis

Data were presented as mean ± standard deviation and analyzed using Stata (version 15, Stata Corp LLC, College Station, TX, USA) and RevMan (version 5.4, Cochrane Collaboration, Oxford, United Kingdom). Effect sizes were calculated using standardized mean differences (SMDs) with 95% confidence intervals. Statistical consistency was ensured by defining effect size directionality: A negative SMD indicates performance improvement for COD time, and a positive SMD indicates improvement for jump distance. SMDs were interpreted as: trivial (SMD <0.20), small (SMD 0.20–0.60), moderate (SMD 0.61–1.20), large (SMD 1.21–2.00), and very large (SMD >2.00) (Hedges, 1985). Between-study heterogeneity was assessed using the I^2^ statistic, with values interpreted as: low (<25%), moderate (25%–75%), and high (>75%) (Nakagawa et al., 2017). Publication bias was evaluated using funnel plots (Peters et al., 2008) and Egger’s regression test (Egger et al., 1997). If Egger’s test indicated potential bias (p < 0.05), the trim-and-fill method was used to adjust effect sizes (Peters et al., 2007). Sensitivity analyses were conducted to test the robustness of the results, and subgroup analyses were performed on the reported outcomes.

## Results

### Study selection

**FIGURE 1:**
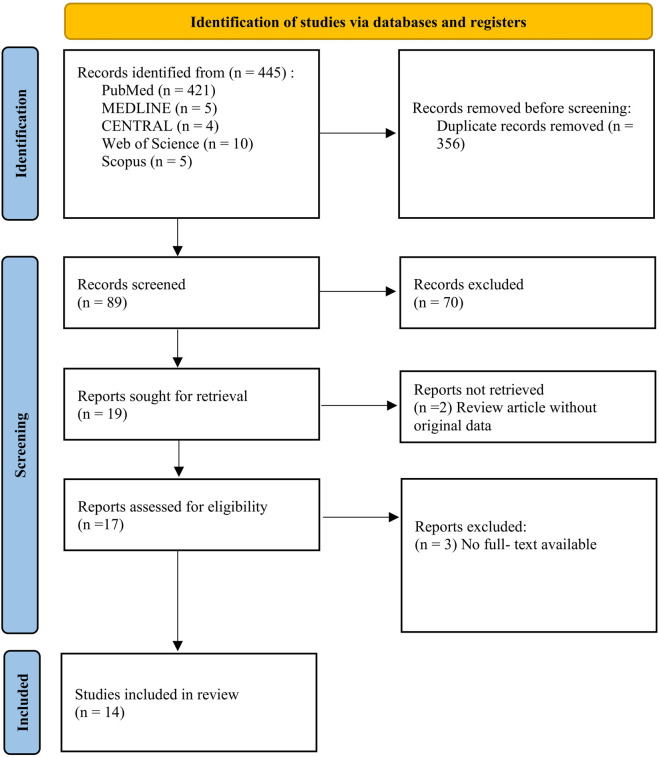
PRISMA flowchart of study selection.

The study selection process is shown in the PRISMA flow diagram (Figure 1). The initial search identified 445 records, of which 356 duplicates were removed. After the removal of duplicates, 89 records were screened. Title and abstract screening excluded 23 reviews and meta-analyses, and 47 irrelevant studies. Seventeen articles underwent full-text review. Two studies were excluded due to unretrievable data, leaving 14 studies for the final quantitative synthesis.

### Study characteristics

The analysis included 14 studies comprising 337 participants (47 females vs. 290 males) aged 12–32 years (Table 3). Participant populations consisted of soccer players (5 studies) (Impellizzeri et al., 2008; Pereira et al., 2023a; Pereira et al., 2023b; Ramirez-Campillo et al., 2020; Zhang et al., 2024), volleyball players (3 studies) (Ahmadi et al., 2021; Sharma and Chaubey, 2013; Yu et al., 2025), and individual studies of basketball (Ozen et al., 2020), tennis (Fernandez-Fernandez et al., 2024), handball (Hammami et al., 2020), and taekwondo (Xie et al., 2025) athletes. Two additional studies examined university students (Meena and Mathur, 2024) and healthy male participants (Arazi et al., 2014), respectively. Experimental groups completed plyometric, sprint, or combined training on sand surfaces, while control groups performed matching training on alternative surfaces. Session durations ranged from 10 to 120 min. Training was typically conducted 3 times per week (range: 1–3 sessions) over 4–12 weeks, with 6–8 weeks representing the most common intervention period.

**TABLE 3:** Characteristics of the studies included in this meta-analysis.

Study	Group	N	Age	Sex	Gender	Intervention	RPE/sRPE	Muscle soreness	Duration	Freq	Time (min)	Outcome
Meena and Mathur, 2024	Land	15		M	Healthy college students	COD and jump training			12	3		T-test, SLJ
	Sand	15										
Hammami et al., 2020	Wood	10	16.2 ± 0.6	M	Junior handball players	Plyometric training			7	3	45	T-test, modified illinois test
	Sand	11	16.4 ± 0.5									
Xie et al., 2025	Floor mat	20		M	Novice taekwondo students	Physical training			8	3	60	T-test, SLJ
	Sand	20										
Arazi et al., 2014	Land	7	20.5 ± 0.3	M	Healthy men	Depth jump training. 5 sets × 20 reps			6	2		T-test, SLJ
	Sand	7	20.7 ± 0.5									
Ramirez-Campillo et al., 2020	Grass	8	12.6 ± 1.8	M	Soccer players	Jump drills 3–25 sets × 5–15 reps			8	2	10–15	COD speed(S)
	Sand	8	12.9 ± 1.2									
Ahmadi et al., 2021	Wood	9	22.7 ± 2.6	F	Volleyball players	Plyometric training. 72–120 contacts/session (2 sets × 6 reps)			8	3	40	T-test
	Sand	8	23.5 ± 2.8									
Pereira et al., 2023a	Grass	8	18.5 ± 0.6	M	Elite young soccer players	Sprint and jump training 2–4 sets × 4–6 reps (jumps); 1–6 sets × 10–15 m (Sprints)	Grass = Sand		8	1	20–30	SJ, CMJ
	Sand	7										
Impellizzeri et al., 2008	Grass	18	25 ± 7	M	Soccer players	Plyometric training 3–25 sets × 5–15 reps		Sand < Grass	4	3		SJ, CMJ
	Sand	19										
Ozen et al., 2020	Wood	6	17.6 ± 0.5	M	Basketball players	Plyometric training 168–252 contacts/3 sets × 3–12 reps			6	3		SLJ, box drill agility, CMJ
	Sand	6										
Sharma and Chaubey, 2013	Court floor	15	16–19	M	Volleyball players	Plyometric Training. 2–4 sets × 6–13 reps			6	2-2-3-2-3–3		SLJ
	Sand	15										
Pereira et al., 2023b	Grass	12	18.3 ± 0.5	M	Soccer players	Sprint and jump training. 2–3 sets × 6–10 reps (jumps); 2–6 sets × 10–15 m (Sprints)			6	2	50	CMJ, SJ, zigzag COD speed tests
	Sand	12										
Jaime et al., 2024	Hard	14	16.5 ± 0.4	M	Tennis players	Sprint and jump training 2–3 sets × 5–10 reps	Grass = Sand	Sand > Hard	6	2		Modified 5–0-5 test, CMJ
	Sand	17	16.2 ± 0.4									
Yu et al., 2025	Sand	10	23.7 ± 2.4	M	Volleyball players	Plyometric training 240–480 contacts/4 sets × 10–20 reps	Grass = Sand		6	2		SLJ
	Land	10	24.1 ± 1.5									
Zhang et al., 2024	Sand	10	21.6 ± 2.3	F	Collegiate soccer players	Sprint interval training program: 5 s all-out running 4 sets × 10 reps	Grass = Sand = Land		7	3	50–52	CMJ, Illinois test
	Land	10	21.9 ± 2.6									
	Grass	10	22.1 ± 2.8									

### Overall effects on COD and jump performance

**FIGURE 2:**
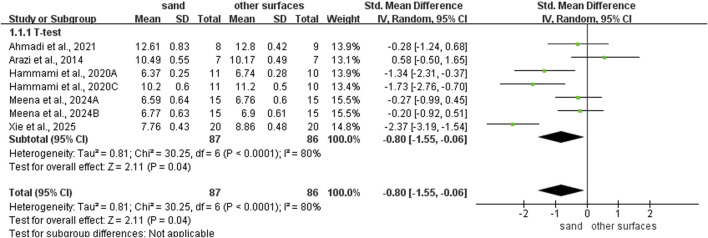
Comparison of training surface on COD performance.

**FIGURE 3:**
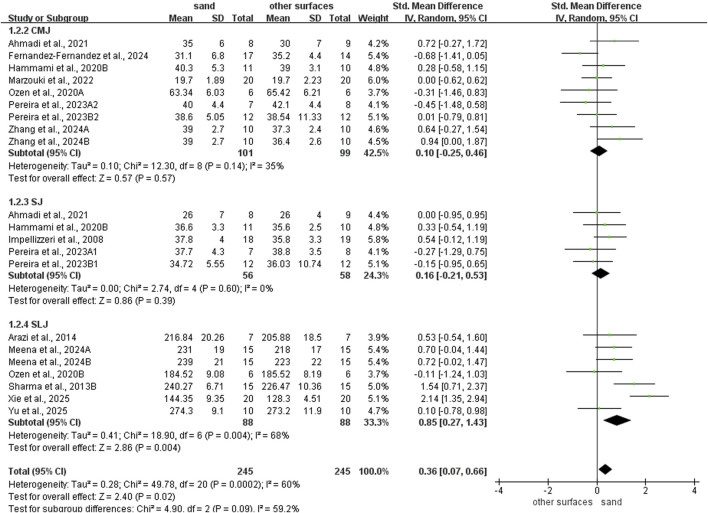
Comparison of training surface on Jump performance.

Sand training demonstrated superior T-test performance compared to other surfaces (SMD: −0.80; 95% CI: −1.55, −0.06; p = 0.04; I^2^ = 80%; Figure 2). Similarly, sand training produced better SLJ results (SMD: 0.85; 95% CI: 0.27, 1.43; p = 0.004; I^2^ = 68%; Figure 3). In contrast, surface type showed no significant effect on CMJ (SMD: 0.10; 95% CI: −0.25, 0.46; p = 0.57; I^2^ = 35%; Figure 3) or SJ (SMD: 0.16; 95% CI: −0.21, 0.53; p = 0.39; I^2^ = 0%; Figure 3) performance.

### Subgroup analysis of COD ability

**FIGURE 4:**
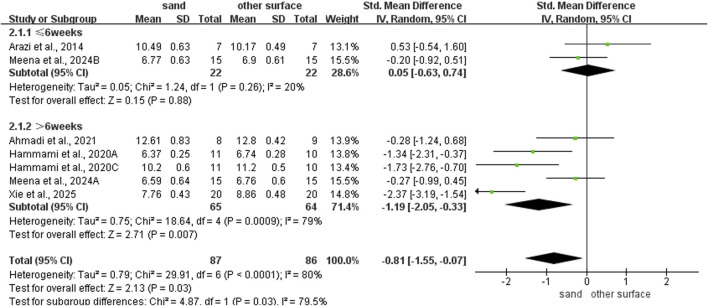
Effect of intervention duration on COD performance with different training surface.

**FIGURE 5:**
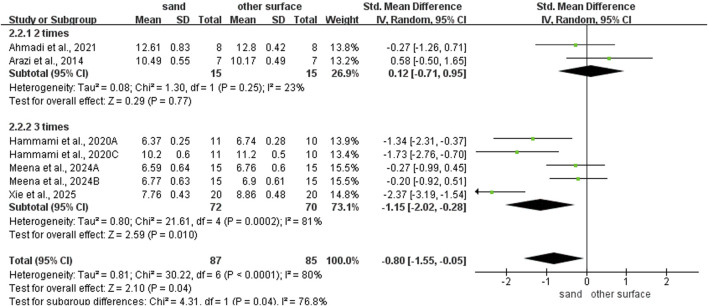
Effect of intervention frequency on COD performance with different training surface.

**FIGURE 6:**
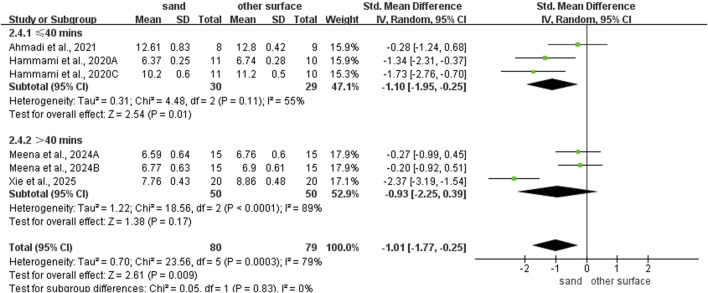
Effect of intervention session on COD performance with different training surface.

**FIGURE 7:**
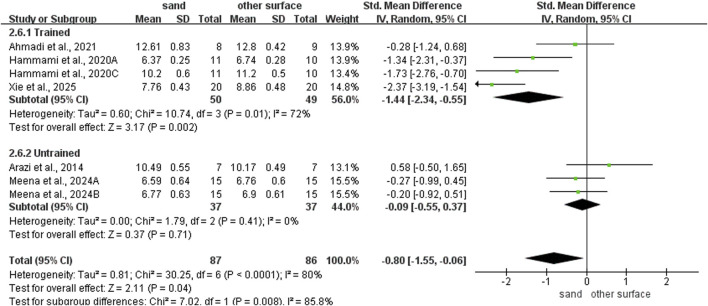
Effect of training background on COD performance with different training surface.

For intervention duration (Figure 4), sand training lasting over > 6 weeks showed better T-test results than other surfaces (SMD: −1.19; 95% CI: −2.05, −0.07; p = 0.007; I^2^ = 79%). No difference was observed for programs lasting 6 weeks or less (SMD: 0.05; 95% CI: −0.63, 0.74; p = 0.88; I^2^ = 20%). For training frequency (Figure 5), sand training with three sessions per week improved T-test performance more than other surfaces (SMD: −1.15; 95% CI: −2.02, −0.28; p = 0.01; I^2^ = 81%). No benefit was found with two weekly sessions (SMD: 0.12; 95% CI: −0.71, 0.95; p = 0.77; I^2^ = 23%). For session duration (Figure 6), sand training lasting ≤40 min produced better T-test results (SMD: −1.10; 95% CI: −1.95, −0.25; p = 0.01; I^2^ = 55%). Longer sessions showed no significant advantage. For training background (Figure 7), experienced participants benefited more from sand training (SMD: −1.44; 95% CI: −2.34, −0.55; p = 0.002; I^2^ = 72%). No benefit was seen in participants without training experience (SMD: −0.09; 95% CI: −0.55, 0.37; p = 0.71; I^2^ = 0%).

### Subgroup analysis of SLJ ability

**FIGURE 8:**
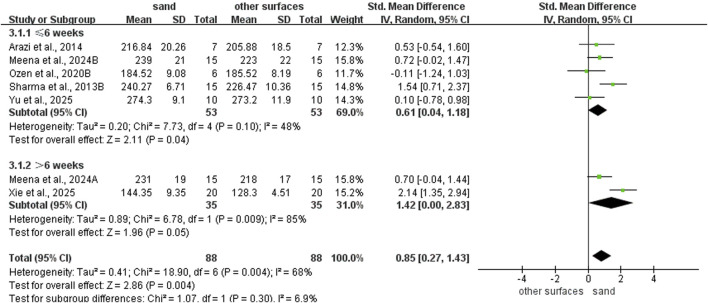
Effect of intervention duration on SLJ performance with different training surface.

**FIGURE 9:**
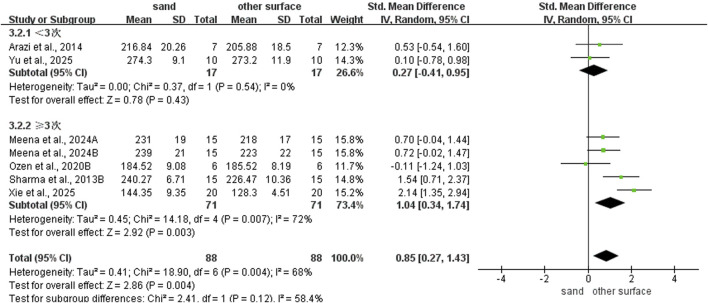
Effect of intervention session on SLJ performance with different training surface.

**FIGURE 10:**
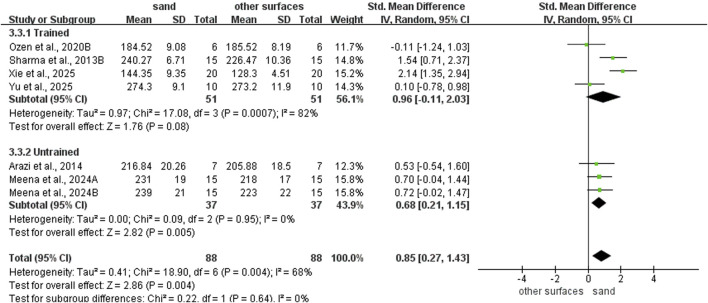
Effect of training background on SLJ performance with different training surface.

Regarding intervention duration (Figure 8), sand training lasting > 6 weeks (SMD: 1.42; 95% CI: 0.00, 2.83; p = 0.05; I^2^ = 85%) and ≤6 weeks (SMD: 0.61, 95% CI: 0.04, 1.18; p = 0.04; I^2^ = 48%) all showed better SLJ results than other surface. However, longer training periods yield superior improvements. For training frequency (Figure 9), sand training performed ≥3 times per week improved SLJ performance more than other surfaces (SMD: 1.04; 95% CI: 0.34, 1.74; p = 0.003; I^2^ = 72%). No benefit was found with fewer than 3 weekly sessions. For training background (Figure 10), participants without training experience had better SLJ results with sand training (SMD: 0.68; 95% CI: 0.21, 1.15; p = 0.005; I^2^ = 0%). No significant difference was seen in experienced participants (SMD: 0.96; 95% CI: −0.11, 2.03; p = 0.08; I^2^ = 82%).

### Methodological quality assessment

Based on the PEDro scale evaluations (Table 4), all 14 included studies exhibited good methodological quality, with scores ranging from six to 7. Blinding of participants, instructors, or outcome assessors was generally not implemented, with one exception that employed a single-blind design (Ozen et al., 2020). This lack of blinding, which is typical in exercise intervention research, represents the primary methodological limitation of the present review.

**TABLE 4:** Methodological quality assessment PEDro.

References (year)	1	2	3	4	5	6	7	8	9	10	11	12
Meena and Mathur, 2024	Y	1	0	1	0	0	0	1	1	1	1	6
Hammami et al., 2020	Y	1	0	1	0	0	0	1	1	1	1	6
Xie et al., 2025	Y	1	0	1	0	0	0	1	1	1	1	6
Ramirez-Campillo et al., 2020	Y	1	0	1	0	0	0	1	1	1	1	6
Marzouki et al., 2022	Y	1	0	1	0	0	0	1	1	1	1	6
Ahmadi et al., 2021	Y	1	0	1	0	0	0	1	1	1	1	6
Pereira et al., 2023a	Y	1	0	1	0	0	0	1	1	1	1	6
Impellizzeri et al., 2008	Y	1	0	1	1	0	0	1	1	1	1	7
Ozen et al., 2020	Y	1	0	1	0	0	0	1	1	1	1	6
Sharma and Chaubey, 2013	Y	1	0	1	0	0	0	1	1	1	1	6
Pereira et al., 2023b	Y	1	0	1	0	0	0	1	1	1	1	6
Fernandez-Fernandez et al., 2024	Y	1	0	1	0	0	0	1	1	1	1	6
Zhang et al., 2024	Y	1	0	1	0	0	0	1	1	1	1	6
Yu et al., 2025	Y	1	0	1	0	0	0	1	1	1	1	6

Pedro’s scale. studies scoring ≥6 are considered high quality, those scoring 4–5 are considered moderate quality, and those scoring ≤3 are considered low quality.

1, eligibility criteria were specified (not included in the total score).

2, subjects were randomly allocated to groups (in a crossover study, subjects were randomly allocated and order in which treatments were received.

3, allocation was concealed.

4, the groups were similar at baseline regarding the most important prognostic indicators.

5, there was blinding of all subjects.

6, there was blinding of all therapists who administered the therapy.

7, there was blinding of all assessors who measured at least one key outcome.

8, measures of at least one key outcome were obtained from more than 85% of the subjects initially allocated to groups.

9, all subjects from whom outcome measures were available received the treatment or control condition as allocated or, where this was not the case, data for at least one key outcome were analyzed by “intention to treat”.

10, the results of between-group statistical comparisons were reported for at least one key outcome.

11, the study provided both point measures and measures of variability for at least one key outcome.

### Publication bias

**FIGURE 11:**
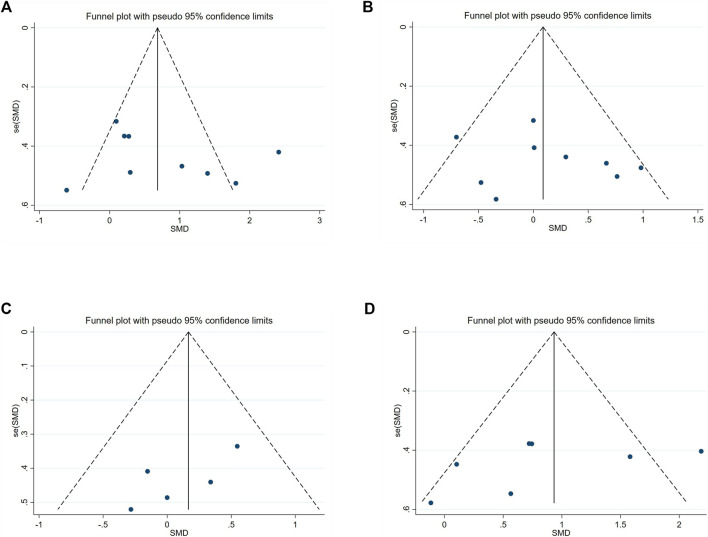
Funnel Plot. **(A)** T-test; **(B)** CMJ; **(C)** SJ; **(D)** SLJ.

No publication bias was detected for any outcome, as shown by the funnel plot (Figures 11A–D) and Egger’s test (Table 5). The results were as follows: T-test (t = 0.25, p = 0.82), CMJ (t = −0.73, p = 0.49), SJ (t = 2.10, p = 0.13) and SLJ (t = −1.09, p = 0.32).

**TABLE 5:** Results of egger’s test for publication bias.

Outcome	Std_Eff	Coef	Std. Err	t	p > |t|	95% CI	
T-test	slope	−0.14	2.71	0.05	0.96	−6.83	7.11
	Bias	1.51	6.13	0.25	0.82	−14.25	17.27
CMJ	slope	0.67	1.07	0.63	0.55	−1.85	3.19
	Bias	−1.79	2.46	−0.73	0.49	−7.61	4.03
SJ	slope	−1.74	0.76	−2.29	0.11	−4.16	0.68
	Bias	3.77	1.80	2.10	0.13	−1.95	9.48
SLJ	slope	3.05	1.97	1.55	0.18	−2.00	8.12
	Bias	−4.94	4.53	−1.09	0.32	−16.60	6.71

p ≤ 0.05 indicates the presence of publication.

### Sensitivity analysis

**FIGURE 12:**
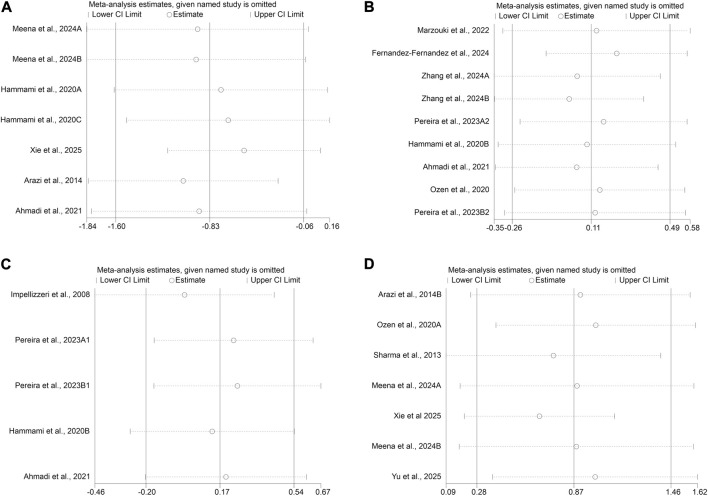
Sensitivity Analysis. **(A)** T-test; **(B)** CMJ; **(C)** SJ; **(D)** SLJ.

A leave-one-out sensitivity analysis was performed using Stata (Figures 12A–D). The results were consistent with the original findings for all tests. The T-test showed similar results (new SMD: −0.83; 95% CI: −1.60, −0.06 vs. original SMD: −0.76; 95% CI: −1.48, −0.12). Similar consistency in SMD was observed for CMJ (0.11 vs. 0.10), SJ (0.16 vs. 0.16), and SLJ (0.87 vs. 0.85). The effects of sand training were found to be robust across all analyses.

## Discussion

This systematic review aimed to explore the impact of different training surface types on COD ability and jump performance, with a focus on identifying dose-response relationships. The findings suggest that sand training is more effective than firm surface training for improving COD and SLJ performance. However, no significant differences were observed for SJ or CMJ performance between surface types. These results contribute to the growing body of evidence on the efficacy of sand training, offering practical insights for designing sand-based training programs.

### COD

Previous meta analyses indicate that training to enhance COD ability, including plyometrics, sprints and combined protocols, are conventionally performed on hard surfaces such as grass and rubber courts to maximize the utility of the stretch-shortening cycle (Nygaard Falch et al., 2019). However, owing to the unique mechanical properties of compliant surfaces, sand has been increasingly investigated as an alternative training modality (Binnie et al., 2014). The current findings align with existing literature. Gastón R (2025) reported that sand training significantly reduces COD completion times in team sport athletes (Sanchez-Ottado et al., 2025). Most interventions in this meta-analysis utilized plyometric or combined running-jumping protocols. Notably, the interventions in our research were not limited to plyometrics but also utilized combined sprints and jump protocols. (Fernandez-Fernandez et al., 2024; Meena and Mathur, 2024; Pereira et al., 2023a; Zhang et al., 2024). The training benefits associated with sand surfaces can be attributed to their mechanical properties. The unstable nature of sand limits elastic energy restitution, requiring increased muscular effort during extended ground contact period (Bauer et al., 1990). This leads to higher muscular activation and greater energy expenditure, promoting neural adaptations such as improved intermuscular coordination and enhanced rate of force development (Aagaard et al., 1985; Sheppard and Young, 2006). These adaptations are particularly beneficial for the rapid acceleration deceleration demands of COD tasks (Negrete and Brophy, 2000). Performance in tests such as the T-test critically depends on the ability to generate force quickly during stance phases (Sheppard and Young, 2006). Sand training appears to improve both eccentric braking capacity and concentric propulsion, thus enhancing COD efficiency (Young et al., 1995).

Notably, effective COD performance depends heavily on horizontal force application (Ramirez-Campillo et al., 2018). Supporting this, Dello Iacono (2017) and Moran (2021) found that horizontal jump training improves COD ability more than vertical jump training (Dello et al., 2017; Moran et al., 2021). This advantage is attributed to the greater horizontal force and shorter ground contact times seen in faster athletes during direction changes (Dos'Santos et al., 2020). Sand training may provide natural resistance for horizontal force development.

This study found that sand training for over 6 weeks, with 3weekly sessions under 40 min, produced better T-test results than other surfaces. Previous reviews have shown that 2-3 sessions per week for 6–8 weeks improve COD ability (Asadi et al., 2016). Short term plyometric training helps youth and amateur athletes, but elite athletes need longer than 6–7 weeks to improve (Slimani et al., 2016). These findings agree with our results. Longer training gives more time for neuromuscular adaptation (Slimani et al., 2016).

Training background also influences results. Individuals with prior training experience showed greater improvements in COD with sand training. The T-test involves various movements like sprinting and shuffling, and trained individuals tend to move faster during direction changes (Wheeler and Sayers, 2010). Sun (2025) confirmed that trained adults have better T–test scores (SMD = - 0.41) (Sun et al., 2025). Together, these findings suggest that longer sand training works better for improving COD ability in athletes.

### Jump performance

Our study found that sand training significantly enhances SLJ performance. This finding is consistent with the meta-analysis by Gastón R et al. (2025), which confirmed that sand training improves SLJ by enhancing neuromuscular adaptations (Sanchez-Ottado et al., 2025). Training surfaces affect horizontal and vertical jumps differently due to their distinct biomechanics. The SLJ is a slow stretch-shortening cycle (SSC) movement with a low takeoff angle (<45°) (Zazulak et al., 2007). It relies more on lower body concentric power than on stored elastic energy (Harry et al., 2021). The sand surface absorbs some elastic energy during takeoff, and extra horizontal force must be produced to overcome this resistance (Lejeune et al., 1998). This overload training strengthens concentric power, thereby improving SLJ performance (Ramlan et al., 2018). Horizontal jumping requires more neuromuscular coordination than vertical jump (Brull-Muria and Beltran-Garrido, 2021; Mann et al., 2021; Rodríguez-Perea et al., 2023). Core muscle activation plays a critical role in trunk stability and force transfer (Brull-Muria and Beltran-Garrido, 2021; Rodríguez-Perea et al., 2023). This affects the speed of the center of mass at takeoff, which ultimately determines jump distance (Brull-Muria and Beltran-Garrido, 2021; Rodríguez-Perea et al., 2023). Furthermore, as an unstable and high resistance training surface, sand not only engages more stabilizing muscles but also enhances neural drive and force output by promoting an external focus of attention (Kibele et al., 2014; Meena and Mathur, 2024). This combination further optimizes performance in the SLJ task.

However, one study found that drop jump training on sand resulted in smaller improvements in SLJ compared to training on firm surfaces (Arazi et al., 2014). This contrasts with our findings, which may be explained by differences in movement types. Drop jumps emphasize rapid SSC with brief ground contacts, while horizontal jumping focuses on concentric power development in a slower SSC context (Bobbert et al., 1987; Harry et al., 2021). The added resistance of sand likely enhances concentric push-off power (Ramlan et al., 2018). Therefore, sand training should be tailored to the type of movement and specific training goals.

While this study and Pereira et al. observed similar CMJ and SJ improvements across surfaces (Pereira et al., 2021). However, the mechanisms behind these improvements are different (Pereira et al., 2021). The SJ primarily measures concentric strength, as it does not involve a pre-stretch (Hasson et al., 2004; McGuigan et al., 2006). On sand, less elastic energy is utilized, and ankle force is more limited, requiring greater concentric force. This explains why sand training is more effective for improving SJ (Giatsis et al., 2004). In contrast, CMJ performance heavily relies on the pre-stretch effect (Cavagna et al., 1968; Kubo et al., 1985). Hard surface training uses the eccentric phase better, making it more effective for CMJ (32). These different mechanisms may complement or offset each other, resulting in similar jump performance improvements (Ramlan et al., 2018). In contrast, muscle soreness is significantly reduced with sand training. This finding offers practical value for high volume training periods or recovery phases (Fernandez-Fernandez et al., 2024; Markovic and Mikulic, 2010). Nonetheless, findings are not entirely consistent. One study involving soccer players found that sand training improved SJ more effectively, while grass training enhanced CMJ performance (Impellizzeri et al., 2008). In summary, sand and hard surfaces improve jump performance through different mechanisms. With the effectiveness depending on the type of jump. Future studies should use magnetoencephalography and ultrasound techniques to explore these mechanisms further, particularly in different athletes and training contexts.

This study found that sand training with more than 3 weekly sessions for 4–6 weeks produced better SLJ results than training on other surfaces. Previous reviews have shown that two to 3 sessions per week for 4–8 weeks can improve jump ability, reaching a level similar to that of hard-surface training (Pereira et al., 2021). Notably, untrained individuals showed greater SLJ improvement on sand than on hard surfaces. In contrast, trained individuals improved similarly on both surfaces. These findings are consistent with previous research. People without training experience adapt more quickly in muscle activation. However, athletes with long term training need more time for lower limb adaptation (Gorostiaga et al., 2006). It is worth noting that only two studies in this analysis examined long-term training, with durations of 8 and 12 weeks (Meena and Mathur, 2024; Xie et al., 2025). Most studies used shorter programs, typically up to 6 weeks. Consequently, the long-term effects of sand training remain incompletely characterized.

### Limitations

This meta-analysis has several limitations. 1) Only 14 studies were included, and some studies measured other abilities, such as the Illinois Agility Test, 505 test, Block jump, and Spike jump. However, these measures could not be included in the analysis due to the small number of studies available. 2) The reporting of training intensity and session timing was insufficient in most studies. Specifically, information regarding jump height, contact time or Rating of Perceived Exertion was lacking. This absence restricts the interpretation of neuromuscular adaptations induced by the training stimuli. 3) The comparison was strictly limited to sand versus hard surfaces. Variations within these categories, such as sand depth, natural grass, artificial turf, wood or rubber, were not individually analyzed due to insufficient data. 4) Only two studies had training periods longer than 6 weeks, making it difficult to assess the long-term effects of sand training. 5) Few studies included female athletes or non-athletes, which limits the generalizability of the findings to these populations.

## Conclusion

Sand training is more effective than other surface training in improving COD ability and horizontal jump performance, while eliciting similar improvements in CMJ and SJ. For COD, the most effective training program involved more than 6 weeks of training, with 3 sessions per week, each lasting no more than 40 min. For horizontal jump performance, the most effective training program included at least 3 sessions per week for 4–6 weeks. Additionally, individuals with training experience showed more significant improvements in COD ability, while those without training experience demonstrated greater improvements in horizontal jump performance. Consequently, coaches are encouraged to strategically incorporate plyometrics, sprint interval training and combined training on sand into weekly training plans, tailored to specific performance goals and training background of the athletes.

## Data Availability

The data analyzed in this study is subject to the following licenses/restrictions: The datasets utilized for the meta-analysis in this study are not provided in the manuscript or supplementary material due to their derivative nature from published articles. However, all data underlying the findings are fully available from the original studies cited in the reference list. Requests to access these datasets should be directed to Tingting Wang; 2023210118@bsu.edu.cn.
