# Correction: Dose-response relationships of sand training compared to other surface training in improving change of direction and jump performance: a systematic review and meta-analysis

**DOI:** 10.3389/fphys.2026.1808452

**Published:** 2026-03-12

**Authors:** Tingting Wang, Haiting Zhai, Hao Yan, Yuping Zhou, Zhi Li, Hongwen Wei, Qian Geng

**Affiliations:** 1 Beijing Sport Science Institute, Beijing, China; 2 Key Laboratory for Performance Training and Recovery of General Administration of Sport, Beijing Sport University, Beijing, China; 3 College of Strength and Conditioning Training, Beijing Sport University, Beijing, China; 4 Sports Coaching College, Beijing Sports University, Beijing, China; 5 Naval Aviation University, Yantai, China; 6 Zhejiang College of Construction, Hangzhou, China; 7 University of Macau, Macao, China; 8 School of Continuous Education, Beijing Sport University, Beijing, China

**Keywords:** change of direction, firm, sand, standing long jump, surface training, systematic review

Affiliation “College of Strength and Conditioning Training, Beijing Sport University, Beijing, China” was omitted for authors Tingting Wang, Hao Yan, and Hongwen Wei. This affiliation has now been added for author Tingting Wang, Hao Yan, and Hongwen Wei as **Affiliation 3**. The remaining affiliations have subsequently been renumbered (affecting authors Haiting Zhai, Yuping Zhou, and Zhi Li).

Affiliation “Zhejiang College of Construction, Hangzhou, China” was erroneously given as “Zhejiang College of Construction, Beijing, China.”

The minus signs (−) before some of the data points were omitted, resulting in an incorrect reversal of the positive and negative values (e.g., values “0.80” and “0.21” should be preceded by a minus symbol). A correction has been made to the section Results, Overall effects on COD and jump performance, Paragraph 1:

“Sand training demonstrated superior T-test performance compared to other surfaces (SMD: −0.80; 95% CI: −1.55, −0.06; p = 0.04; I^2^ = 80%; **Figure 2**). Similarly, sand training produced better SLJ results (SMD: 0.85; 95% CI: 0.27, 1.43; p = 0.004; I^2^ = 68%; **Figure 3**). In contrast, surface type showed no significant effect on CMJ (SMD: 0.10; 95% CI: −0.25, 0.46; p = 0.57; I^2^ = 35%; **Figure 3**) or SJ (SMD: 0.16; 95% CI: −0.21, 0.53; p = 0.39; I^2^ = 0%; **Figure 3**) performance.”

The minus signs (−) before some of the data points were omitted, resulting in an incorrect reversal of the positive and negative values (e.g., values “1.19”, “2.05”, “0.63”, “1.15”, “2.02”, “0.71”, “1.10”, “1.95”, “1.44”, “2.34”. “0.09”, and “0.55” should be preceded by a minus symbol). A correction has been made to the section Results, Subgroup analysis of COD ability, Paragraph 1:

“For intervention duration (**Figure 4**), sand training lasting over >6 weeks showed better T-test results than other surfaces (SMD: −1.19; 95% CI: −2.05, −0.07; p = 0.007; I^2^ = 79%). No difference was observed for programs lasting 6 weeks or less (SMD: 0.05; 95% CI: −0.63, 0.74; p = 0.88; I^2^ = 20%). For training frequency (**Figure 5**), sand training with three sessions per week improved T-test performance more than other surfaces (SMD: −1.15; 95% CI: −2.02, −0.28; p = 0.01; I^2^ = 81%). No benefit was found with two weekly sessions (SMD: 0.12; 95% CI: −0.71, 0.95; p = 0.77; I^2^ = 23%). For session duration (**Figure 6**), sand training lasting ≤40 min produced better T-test results (SMD: −1.10; 95% CI: −1.95, −0.25; p = 0.01; I^2^ = 55%). Longer sessions showed no significant advantage. For training background (**Figure 7**), experienced participants benefited more from sand training (SMD: −1.44; 95% CI: −2.34, −0.55; p = 0.002; I^2^ = 72%). No benefit was seen in participants without training experience (SMD: −0.09; 95% CI: −0.55, 0.37; p = 0.71; I^2^ = 0%).”

The minus signs (−) before some of the data points were omitted, resulting in an incorrect reversal of the positive and negative values (e.g., value “0.11” should be preceded by a minus symbol). A correction has been made to the section Results, Subgroup analysis of SLJ ability, Paragraph 1:

“Regarding intervention duration (**Figure 8**), sand training lasting >6 weeks (SMD: 1.42; 95% CI: 0.00, 2.83; p = 0.05; I^2^ = 85%) and ≤6 weeks (SMD: 0.61, 95% CI: 0.04, 1.18; p = 0.04; I^2^ = 48%) all showed better SLJ results than other surface. However, longer training periods yield superior improvements. For training frequency (**Figure 9**), sand training performed ≥3 times per week improved SLJ performance more than other surfaces (SMD: 1.04; 95% CI: 0.34, 1.74; p = 0.003; I^2^ = 72%). No benefit was found with fewer than 3 weekly sessions. For training background (**Figure 10**), participants without training experience had better SLJ results with sand training (SMD: 0.68; 95% CI: 0.21, 1.15; p = 0.005; I^2^ = 0%). No significant difference was seen in experienced participants (SMD: 0.96; 95% CI: −0.11, 2.03; p = 0.08; I^2^ = 82%).”

The minus signs (−) before some of the data points were omitted, resulting in an incorrect reversal of the positive and negative values (e.g., values “0.83”, “1.60”, “0.76”, and “1.48” should be preceded by a minus symbol). A correction has been made to the section Results, Sensitivity analysis, Paragraph 1:

“A leave-one-out sensitivity analysis was performed using Stata (**Figures 12A–D**). The results were consistent with the original findings for all tests. The T-test showed similar results (new SMD: −0.83; 95% CI: −1.60, −0.06 vs. original SMD: −0.76; 95% CI: −1.48, −0.12). Similar consistency in SMD was observed for CMJ (0.11 vs. 0.10), SJ (0.16 vs. 0.16), and SLJ (0.87 vs. 0.85). The effects of sand training were found to be robust across all analyses.”

The original article has been updated.

